# Neuroimmunomodulation of adrenoblockers during liver cirrhosis: modulation of hepatic stellate cell activity

**DOI:** 10.1080/07853890.2022.2164047

**Published:** 2023-02-24

**Authors:** Mariana Yazmin Medina Pizaño, María de Jesús Loera Arias, Roberto Montes de Oca Luna, Odila Saucedo Cárdenas, Javier Ventura Juárez, Martin Humberto Muñoz Ortega

**Affiliations:** a Histology Department, Faculty of Medicine, Autonomous University of Nuevo León, Monterrey, México; b Department of Morphology, Autonomous University of Aguascalientes, Aguascalientes, México; c Department of Chemistry, Autonomous University of Aguascalientes, Aguascalientes, México

**Keywords:** Cirrhosis, hepatic cells, stellate cells, sympathetic nervous system, neuroimmunomodulation, adrenoblockers

## Abstract

The sympathetic nervous system and the immune system are responsible for producing neurotransmitters and cytokines that interact by binding to receptors; due to this, there is communication between these systems. Liver immune cells and nerve fibres are systematically distributed in the liver, and the partial overlap of both patterns may favour interactions between certain elements. Dendritic cells are attached to fibroblasts, and nerve fibres are connected via the dendritic cell-fibroblast complex. Receptors for most neuroactive substances, such as catecholamines, have been discovered on dendritic cells. The sympathetic nervous system regulates hepatic fibrosis through sympathetic fibres and adrenaline from the adrenal glands through the blood. When there is liver damage, the sympathetic nervous system is activated locally and systemically through proinflammatory cytokines that induce the production of epinephrine and norepinephrine. These neurotransmitters bind to cells through α-adrenergic receptors, triggering a cellular response that secretes inflammatory factors that stimulate and activate hepatic stellate cells. Hepatic stellate cells are key in the fibrotic process. They initiate the overproduction of extracellular matrix components in an active state that progresses from fibrosis to liver cirrhosis. It has also been shown that they can be directly activated by norepinephrine. Alpha and beta adrenoblockers, such as carvedilol, prazosin, and doxazosin, have recently been used to reverse CCl_4_-induced liver cirrhosis in rodent and murine models.

**KEY MESSAGES**
- Neurotransmitters from the sympathetic nervous system activate and increase the proliferation of hepatic stellate cells.
- Hepatic fibrosis and cirrhosis treatment might depend on neurotransmitter and hepatic nervous system regulation.
- Strategies to reduce hepatic stellate cell activation and fibrosis are based on experimentation with α-adrenoblockers.

## Neuroimmunomodulation

The modulating effect of the nervous system on immunological processes is known as neuroimmunomodulation. This modulation considers the reciprocal communication of the immune and neurological systems. Because immune cells have neurotransmitter receptors (including those for norepinephrine and acetylcholine) and lymph nodes are innervated by sympathetic nervous system (SNS) fibres, neuroimmunomodulation is possible [1]. These innervating fibres impact immune cell migration and proliferation, promoting neuroimmunomodulation. The vagus nerve, which inhibits cytokine generation in peripheral monocytes *via* the alpha-7 nicotinic acetylcholine receptor, is a recently discovered alternative approach to neuroimmunomodulation [2]. Since the inflammatory response is the basis of severe chronic disease pathogenesis, such as cancer and coronary heart disease, the neuroimmunomodulating function of the vagus nerve may have clinical significance. Therefore, it is postulated that vagal activity might modulate disease progression [3,4], an issue currently under investigation. The cerebral hemispheres’ influences on peripheral immunity is another way that neuroimmunomodulation manifests. According to studies, the right hemisphere has immunosuppressive effects, while the left hemisphere has immune-potentiating effects [5,6]. Since inhibiting beta-adrenergic receptors eliminated disparities in the immune response between the hemispheres, these effects were observed in animals and humans and mediated by the sympathetic response.

Although a change in activity from the left to the right hemisphere during stressful times was associated with an increased incidence of reported sickness [7], the neuroimmunomodulatory effects of the hemispheres may have clinical relevance. Additionally, in a matched prospective design, regardless of factors, those with right hemisphere lateralization had a substantially higher chance of reporting the common cold than those with left hemisphere lateralization [8].

Since the SNS, vagal nerve activity, and hemispheric lateralization are all connected to psychological factors, immunity, and the threat of liver injury, neuroimmunomodulation plays a key role in the function of medicine. It may help explain how psychological factors affect the likelihood of developing a disease. Understanding these neuromodulator interactions is key to potentially prevent or treat liver illnesses such as cirrhosis.

## Sympathetic nervous system

The sympathetic nervous system consists of multiple pathways that interact with different organ systems in multiple ways. The thoracic and lumbar portions of the spinal cord (T1 to L2) are the source of the preganglionic neurons of the SNS. The cell bodies are symmetrically and bilaterally distributed in four areas of the gray matter of the spinal cord [9]. The first-order neurons of the sympathetic nervous system (SNS) synapse with postsynaptic neurons within the sympathetic ganglia, but they are shorter than the parasympathetic nervous system. Acetylcholine is the neurotransmitter at this junction, similar to the parasympathetic nervous system (PNS). The nicotinic and muscarinic receptors in acetylcholine are activated.

In contrast to the sympathetic innervation of sweat glands and arrector pili—the small muscles attached to hair follicles, which use acetylcholine as their postganglionic neurotransmitter—these postganglionic neurons travel to their effector sites and release the neurotransmitters epinephrine (EPI) or norepinephrine (NE) [10]. These neurotransmitters regulate adrenergic receptors. Amid the adrenergic receptors are alpha-1 (coupled to a Gq and activating through the IP3/Ca^2+^ pathway), alpha-2 (coupled to Gi and acting by decreasing the cAMP pathway), and beta-1 and beta-2 (coupled to Gs and working by increasing the cAMP pathway) [11]. Depending on the tissue in which they are situated, adrenergic receptors can either be excitatory or inhibitory.

## Immunomodulatory function of the sympathetic nervous system

### Modulation of immune and inflammatory processes by the sympathetic nervous system

Primary and secondary lymphoid organs have noradrenergic sympathetic innervation. When there is extensive stimulation, NE is released, and immune cells express adrenoceptors. Through the stimulation of these receptors, locally released NE or catecholamines such as epinephrine modify lymphocyte trafficking, circulation, and proliferation and modulate cytokine production and Kupffer cell activity. There is evidence that NE and epinephrine, through the beta(2)-adrenoreceptor-cAMP-protein kinase A pathway, can inhibit the production of type-1 proinflammatory cytokines, such as IL-12, TNF-α, IFN-γ through antigen-presenting cells and helper T cells (Th)1. In contrast, the production of type-2 anti-inflammatory cytokines such as IL-10 and TGF-β is stimulated. Through this mechanism, endogenous catecholamines can systemically cause a selective suppression of Th1 responses and cellular immunity and a Th2 shift toward the domain of humoral immunity [12]. NA activates immune cell functions, primarily NK cells, macrophages, and T and B lymphocytes. In humans, catecholamine injection causes an increase in NK cell migration in the first 2 to 4 h. However, over time, it causes a decline in their functionality and granulocytes to be attracted to specific sites [12]. By preventing neutrophils from releasing lysosomal enzymes and participating in phagocytosis, catecholamines also affect innate immunity [13]. They also prevent the degranulation-related neutrophil respiratory burst at low doses [14].

Additionally, at nanomolar adrenaline concentrations, the production of superoxide and the creation of oxygen radicals is reduced. The stimulation of beta-adrenergic receptors (β-2) is what causes these effects. Contrarily, catecholamines are also known to activate and alter the effects on innate and acquired immunity when they interact with alpha-adrenergic receptors. Beta receptor stimulation also prevents human neutrophil chemotaxis when leukotriene B4 and formyl-methionyl-leucyl-phenylalanine, two potent chemoattractants, are present [12].

Catecholamine synthesis blockers are used to investigate the effects of the SNS. Their function is to cause reversible atrophy of the noradrenergic terminals through a loss of membrane integrity brought on by a reduction in the generation of hydroxyl radicals and hydrogen peroxide [15]. The immune cells that depend on this system’s function are altered because the lymphocytes’ adrenergic receptors are not activated [16]. According to authors [17], afferent nerves to the liver enter the parenchyma *via* the bile duct, hepatic artery, or portal vein. Somatic nerves come from the spinal cord segments T-7 to T-10 and innervate the liver through the celiac ganglion. Parasympathetic fibres come from the vagus nerve and innervate the liver.

The innervation patterns differ in the livers of diverse species, as described in previous research in this issue. The hepatic pedicle and periportal areas of rodents contain a high concentration of nerve fibres of all types. A few are found along the hepatic veins, but only a rare fibre is identified inside the lobules. On the other hand, primates exhibit considerable intralobular innervation [18]. When analysing results from mouse investigations, the simple absence of intralobular innervation creates some difficulties because it suggests that sympathetic nerve fibre expression and neuronal influence on inflammatory processes in the liver are only present in the portal area. Humans, in contrast, express markers of efferent sympathetic fibres that terminate on liver parenchymal cells and extend deep into connective tissue and hepatic lobules [19].

Another concern is the contradiction between the abundance of functional evidence for cholinergic liver innervation [20,21] and the lack of morphological evidence, at least in rodents, for cholinergic intrahepatic nerve fibres [18]. Recent reports on the inflammatory reflex, which is thought to use cholinergic vagal routes to liver macrophages, are even more puzzling by this conundrum [22]. However, the distribution of nerve fibres and immune cells in the liver is not random, and the partial overlap of these patterns may promote interactions between some aspects. Others, however, disagree [17].

## Liver cirrhosis

Cirrhosis was previously defined morphologically as anomalous liver architecture with fibrous bands around regenerative nodules [23]. It is important to emphasize that fibrosis and cirrhosis, frequently used synonymously, are clinically separate entities. It could be argued that fibrosis in and of itself in a pre-cirrhotic liver has little clinical significance because the hepatic reserve is still relatively intact. The increased risk of HCC is linked to liver cirrhosis of all aetiologies with one caveat that some liver illnesses have been shown to raise the risk of HCC in patients who are not yet cirrhotic.

The definition of cirrhosis should incorporate at least three important factors: physiological disruption of the vasculature, which contributes to the emergence of portal hypertension; an alteration in hepatic function, which may ultimately result in decompensated liver disease; and an increased risk of neoplastic transformation, a phenomenon relevant to cirrhosis of all aetiologies; all should be included in the definition of cirrhosis. These variables have a significant clinical impact that contributes to liver-related mortality and morbidity [24].

## Types of cells that influence liver cirrhosis (cell involvement)

### Hepatic stellate cells

**Figure 1.**
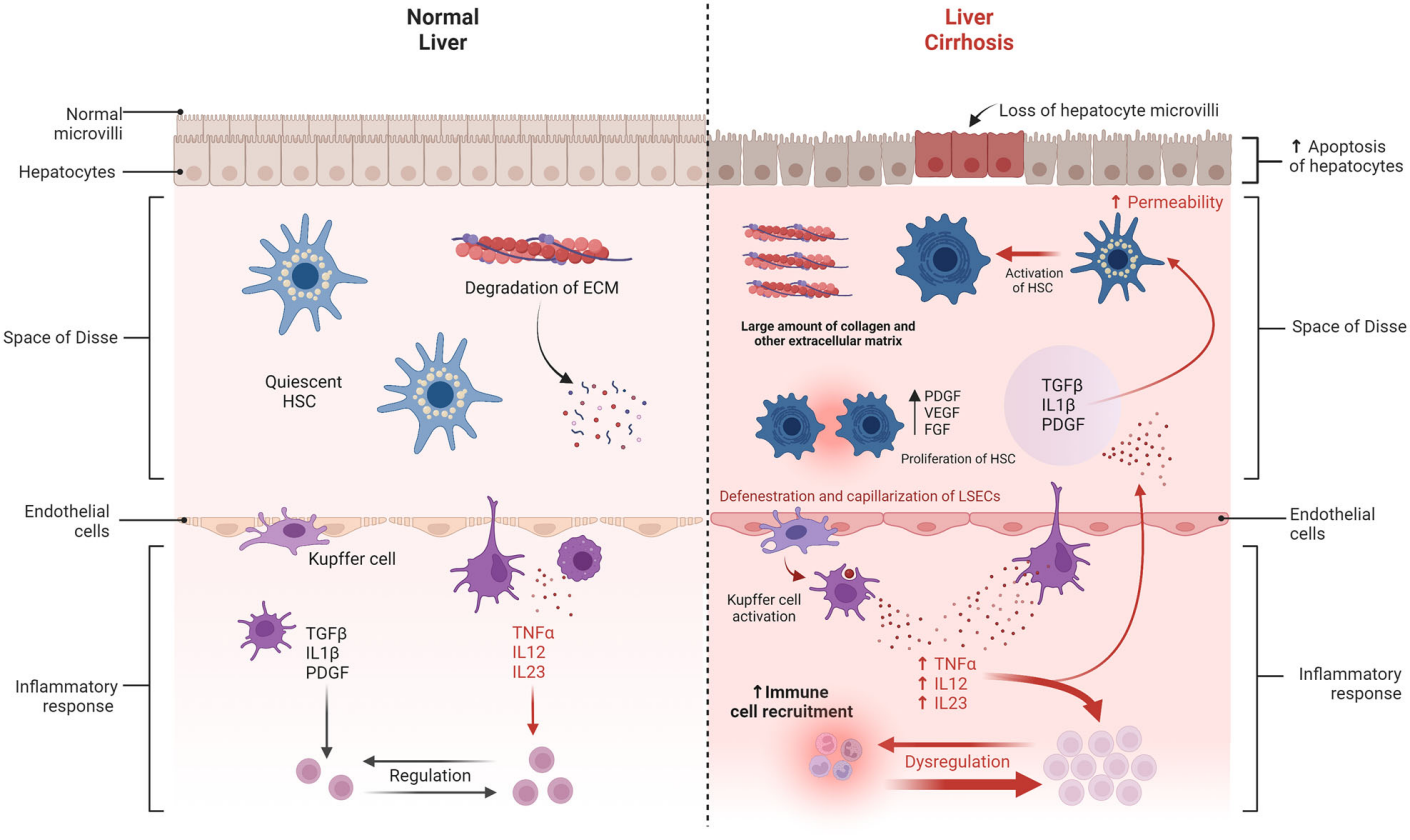
Hepatic stellate cell activation during liver injury and resolution.

HSCs are converted from a quiescent to an activated state by inflammatory cytokines, such as platelet-derived growth factor (PDGF), transforming growth factor (TGF)-β, tumour necrosis factor (TNF)-α, and interleukin (IL-1). HSC activation is a critical step in the progression of liver fibrosis and a key factor in collagen deposition (Figure 1) [25]. Activated HSCs change their phenotype to myofibroblast and initiate the production of collagen and extracellular matrix (ECM) components [26].

HSCs are primarily responsible for fibrosis in zone 3, known as perisinusoidal fibrosis. On the other hand, fibrosis in zone 1, known as portal fibrosis, is associated with the progression of damage in steatohepatitis and viral hepatitis. Periportal fibrosis has not been studied extensively, and it is unclear how and why portal fibrosis develops in chronic liver disease where the initial primary insult is within the lobules. In cholestatic liver disorders, it is common to observe the development of portal fibrosis in response to ductular morphological change. Studies in humans with biliary diseases and animal models of biliary fibrosis have shown that reactive or deteriorating ductal epithelium can express profibrogenic proteins, such as PDGF and TGF-β, and chemotactic proteins that activate both inflammatory cells and fibrogenic cells developing a periportal fibrosis process [27–30].

### Hepatic progenitor cells (HPCs)

HPCs exist in normal liver tissue in the progenitor cell compartment and reside in the canals of Hering in bile ducts. HPCs are referred to as oval cells and possess the potential to differentiate toward the hepatocytic or biliary phenotype. Histologically, these cells adopt the appearance of small epithelial cells with an oval nucleus and scant cytoplasm. They express markers such as OV-6 and chromogranin A. As reservoir cells, HPCs were shown to be activated in a wide range of liver diseases and conditions, such as sub-massive necrosis, chronic viral hepatitis, and fatty liver disease [31].

### Liver sinusoidal endothelial cells

Defenestration of the sinusoidal endothelium and a subendothelial basement membrane are frequent characteristics of the cirrhotic liver [32]. Retinol shortage can activate HSCs, transform them into myofibroblasts with increased ECM synthesis, and ultimately lead to cirrhosis [33]. Because of modifying retinol metabolism, it is thought that defenestration and capillarization of the hepatic endothelium are crucial for the onset of perisinusoidal cirrhosis. According to different studies, the defenestration of the liver sinusoidal endothelial cells, through impairment of substrate exchange, contributes to liver cirrhosis dysfunction [34].

Interleukin-33 (IL-33) expression is upregulated in human and murine hepatic fibrosis. This expression is related to stressed hepatocytes, which trigger the profibrogenic activation of hepatic stellate cells *via* mediators such as IL-13 [35].

Nevertheless, differentiated LSECs can promote regression and prevent the advancement of fibrosis by encouraging the reversal of activated HSCs to quiescence through VEGF-stimulated NO production [36].

### Kupffer cells

Research using animal models has demonstrated that *Kupffer cells* (KCs) play a role in the aetiology of several liver disorders [37]. Liver damage and fibrosis are aggravated by KC-mediated hepatic inflammation [38]. HSC activation and fibrosis development are both aided by KCs. By inducing the expression of PDGF receptors in HSCs, *in vitro* studies have demonstrated that a KC-conditioned media can promote the activation of cultured rat HSCs with enhanced matrix synthesis and cell proliferation [39]. HSCs obtained from rats treated with a high-fat diet and ethanol are stimulated to proliferate and produce collagen by KC-derived TGF-β1 [40]. By degrading collagen type IV, gelatinase released by activated KCs causes the phenotypic change in HSCs (Figure 1) [41]. Inflammation and fibrogenesis are facilitated by KCs’ consumption of apoptotic bodies and the production of death ligands like TNF-α and the Fas ligand [42]. In both normal and fibrotic livers, KCs triggered by β-glucans raise portal pressure by releasing thromboxane A2 [43].

### Hepatocytes

Cirrhosis and other chronic liver diseases can stimulate hepatocyte regeneration in response to compensation or induce apoptosis. Hepatocyte damage results from the release of reactive oxygen species (ROS), profibrotic mediators, HSC activation, and stimulation of myofibroblast effects. As a result of this process, there is tissue inflammation and disease progression [44]. Hepatocyte apoptosis is induced in the early stages of CCl_4_-induced liver injury, followed by constant proliferation, and if it persists, liver cirrhosis develops at a later stage [45]. Matrix metalloproteinases (MMP-2, MMP-3, and MMP-13) and tissue inhibitors of matrix metalloproteinases (TIMP-1 and TIMP-2) are primarily produced by hepatocytes. They are all involved in the aetiology of liver cirrhosis in rats with liver cirrhosis induced by CCl_4_ [46]. Hypoxic hepatocytes become the main source of TGF-β1 in the last fibrotic stage of cirrhosis, aggravating hepatic fibrogenesis [47]. The pathophysiology of cirrhosis has recently been explained uniquely by the discovery that hepatocyte telomere shortening, and senescence can cause fibrotic scarring at the cirrhosis stage [48].

## Role of growth factors and cytokines in liver cirrhosis (humoral involvement)

### PDGF

Of all the polypeptide growth factors, PDGF is the strongest mitogen for HSCs. PDGF-A, PDGF-B, PDGF-C, and PDGF-D are the four members of the PDGF family [49]. In fibrous tissues, PDGF and its receptors are noticeably overexpressed, and the degree of liver fibrosis promotes the activity of these molecules [50]. Numerous factors, including viruses, drugs, or physical harm to the hepatocytes, can cause KCs to produce and release PDGF [51]. PDGF activates corresponding signal molecules and transcription factors upon binding to its receptor on the membrane of HSCs, which in turn causes the activation of its downstream target genes and HSCs [52]. According to research, PDGF increases MMP-2, MMP-9, and TIMP-1 expression and inhibits collagenase activity, which inhibits the degradation of the extracellular matrix (ECM) [49,52].

According to PDGFRβ autophosphorylation and activation of the ERK1/2, C-Jun N-terminal kinase (JNK), p38 mitogen-activated protein kinase (MAPK), and protein kinase (PK)B/Akt pathways, PDGF-B and PDGF-D are potent PDGF isoforms in PDGF receptor (PDGFR)β signalling within HSCs [53]. Although PDGF-D can activate HSCs and has mitogenic and fibrotic effects, it is essential for remodelling the matrix in liver fibrosis [54].

### TGF-β

The strongest known inducer of fibrogenesis in cirrhosis and liver fibrosis is TGF-β. The liver’s HSCs/myofibroblasts, KCs, LSECs, and hepatocytes are the principal producers of TGF-β. The TGF-β1 family includes 3 members. TGF-β1 has been demonstrated essential for developing and maintaining liver fibrosis [55]. TGF-β1 expression increases in the fibrotic liver and culminates at the stage of cirrhosis [47]. By suppressing the expression of MMPs and promoting TIMP, TGF-β1 induces the expression of the matrix-producing genes, prevents the degradation of ECM, and promotes liver fibrosis. This change causes an excessive accumulation of collagenous fibres [56]. Furthermore, it has been demonstrated that TGF-β1 inhibits DNA synthesis and induces apoptosis in hepatocytes. It is thought that tissue loss and a decrease in liver size in cirrhosis are caused by TGF-β1-induced apoptosis [57].

### TNF-α

Kupffer cells, HSC, macrophages, and monocytes are the main producers of TNF-α; these cells have cytotoxic and proinflammatory actions. TNF-α is essential for HSC stimulation and ECM production throughout liver fibrosis [58]. Upregulating the antiapoptotic proteins NF-κB and Bcl-XL and downregulating the proapoptotic factor p53, TNF-α can decrease spontaneous apoptosis of activated rat HSCs [59]. TNF-α can cause HSC apoptosis, indicating that its effects on fibrosis are complicated and contradictory [60]. It has also been shown that TNF-α has an antifibrogenic effect in rat HSCs, the expression of which can decrease glutathione and prevent the production of pro-collagen 1 [61].

### Interferon

In response to viral infection, leukocytes produce IFN-α and IFN-β, and T lymphocytes release IFN-γ after being stimulated by various mitogens and antigens. IFNs have antiviral activity, and their antiviral effects are well known [62]. Even in the absence of viral eradication, IFN-treated patients show a regression of liver fibrosis, demonstrating that IFN has an antifibrotic effect by inducing HSC apoptosis [63]. By inhibiting the TGF-β and PDGF pathways, IFN-β might inactivate HSCs and reduce their synthesis of α-smooth muscle actin (SMA) and collagen [64]. Like this, it has been shown that IFN-γ inhibits HSC activation *via* TGF-β1/Smad3 signalling pathways, hence reducing ECM deposition *in vivo*. IFN-γ treatment for liver fibrosis in rats resulted in less collagen, laminin, fibronectin, and pro-collagen type I being produced and deposited [65].

### Interleukins

Leukocytes were initially found to express a group of cytokines known as ILs; however, it was later discovered that a wide variety of other cells, including CD4 T lymphocytes, monocytes, macrophages, and endothelial cells, were also capable of producing ILs [66]. Pro-fibrotic ILs: In response to liver tissue damage, KCs and SECs can quickly create ILs. As a result of IL-1's direct activation of HSCs and stimulation of their production of MMP-9, MMP-13, and TIMP-1, hepatic fibrogenesis occurs. In contrast, IL-1-receptor-deficient animals show reduced susceptibility to fibrosis development and liver injury [67]. Similarly, it was discovered that IL-1 receptor antagonists prevented dimethylnitrosamine-induced liver fibrosis in rats [68].

Hepatocytes’ production of the pro-steatosis chemokine monocyte chemoattractant protein-1 and macrophages’ Toll-like receptor (TLR4)-dependent amplification of inflammatory signaling were both reported increased by IL-1β [69]. Profibrotic cytokine IL-17 is expressed at higher levels in livers with more fibrosis, suggesting that IL-17 may contribute to the development and persistence of the illness [70]. Studies in mice have demonstrated that IL-17 induces liver fibrosis through various mechanisms, including the promotion of HSCs’ myofibroblast-like change and upregulation of TNFα-, TGF-β, and collagen 1α. These mechanisms depend on the STAT3 signalling pathway and the upregulation of TNF-, TGF-, and collagen 1 [71].

Antifibrogenic ILs: IL-10 is a cytokine that modulates hepatic fibrogenesis and downregulates the proinflammatory response [72]. Exogenous IL-10 was shown to reverse CCl_4_-induced hepatic fibrosis by reducing the production of TGF-β1, MMP-2, and TIMP-1 in a rat model, demonstrating that IL-10 has antifibrotic effects *via* inhibiting HSC activity [73,74].

IL-22 promotes antimicrobial immunity, inflammation, and tissue healing at barrier surfaces. In a mouse model, IL-22 has been demonstrated to cause HSC senescence, limit liver fibrosis, and hasten liver fibrosis resolution after recovery [75].

IL-6 is a pleiotropic cytokine that regulates immunological function, haematopoiesis, and inflammatory pathways. By promoting hepatocyte regeneration through NF-κB signalling and the Ras-MAPK pathway, it can attenuate apoptosis and lessen CCl_4_-induced acute and chronic liver injury as well as fibrosis [76]. Improved liver injury following fibrosis results from pretreating the fibrotic liver with IL-6, which also enhances the hepatic microenvironment and prepares it for mesenchymal stem cell transplantation [77].

## Adrenoreceptors on immune cells and liver stellate cells

**Figure 2.**
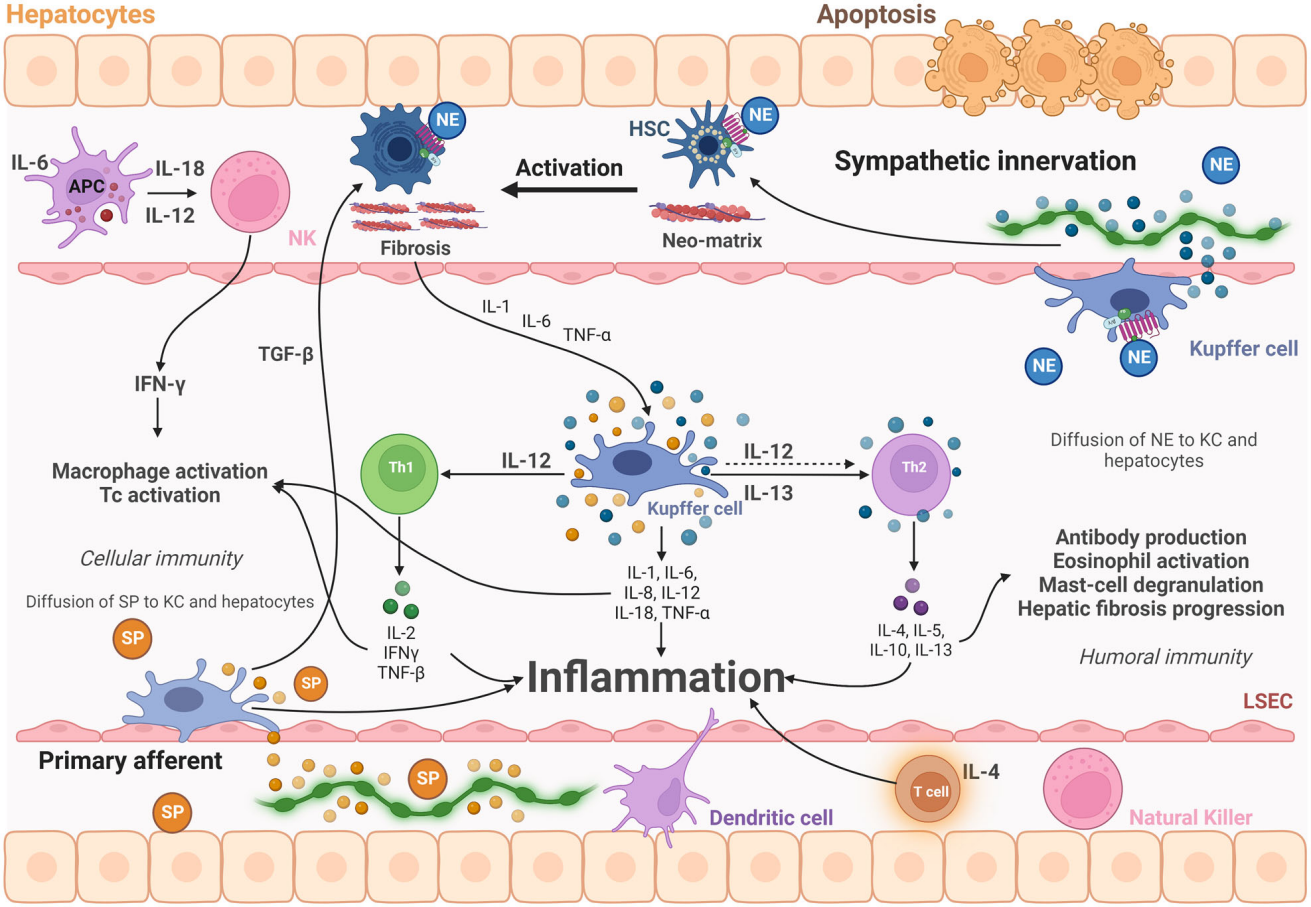
Modulation by neurotransmitters, neuropeptides and adrenoreceptors of cells and cytokines of the immune response during the development of liver fibrosis. KCs and hepatocytes are distant from these nerve fibres; however, diffusion of norepinephrine along sinusoids may be efficient enough to modulate KCs and activate apoptosis in hepatocytes.

As described above, there is an anatomical mismatch between the distribution of nerve fibres to KCs and hepatocytes, the non-neuronal component of the liver. T cells and dendritic cells are located in the periportal area, so they can easily contact nerve fibres and be influenced by neurotransmitters. Most KCs, and hepatocytes, except those found in the periphery, are far from the nerve fibres due to their intralobular location. Therefore, it is believed that in rodent models of hepatitis treated with neural modulation, KCs and hepatocytes may be in contact with neurotransmitters released by periportal innervation. It is proposed that the diffusion of norepinephrine or substance P (SP) along the sinusoids into the lobes may be efficient enough to modulate KC function toward an inflammatory profile and hepatocyte apoptosis (Figure 2). However, this usually occurs in the lobes [78].

The discovery that the immunosuppressive effect is truly caused by α-adrenergic stimulation is supported by the inhibition of the T cell response with the α-blocker, phentolamine. Additionally, it was discovered that the relevant receptor belongs to the α2-subtype employing selective subtype agonists [79]. When β-receptors were concomitantly blocked with propranolol, long-term *in vivo* treatment with noradrenaline or adrenaline resulted in a substantial decrease of the *in vitro* reactivity of peripheral blood T cells [80].

A study demonstrated that HSCs express important enzymes for catecholamine biosynthesis and generate NE and other catecholamines. Furthermore, when normal HSCs are cultured with α- or β-adrenoceptor antagonists, increasing HSC numbers are considerably suppressed, indicating that HSCs use catecholamines to autoregulate their proliferation. Studies using inhibitors indicate that kinases that support cell survival and proliferation must be activated for NE to exert its trophic effects. The activation of comparable signalling pathways by leptin, another substance that stimulates HSC development, makes this study intriguing [81].

The functional expression of α/β-adrenoceptors and neuropeptide Y receptors (NPY), which are increased in the livers of individuals with cirrhotic NAFLD, is demonstrated in a study of activated human primary hepatic stellate cells (hHSC). hHSC produce and release NE/EPI in culture, which is necessary for healthy basal growth and survival. Exogenous NE/EPI and NPY stimulated hHSC proliferation in a dose-dependent *via* p38 MAP, PI3K, and MEK signalling. Without the involvement of the pro-fibrogenic cytokines leptin, IL-4, and IL-13, or the antifibrotic cytokine IL-10, NE, and EPI, but not NPY-enhanced expression of collagen-1α2 *via* TGF-β [82].

## Proliferation of hepatic stellate cells by norepinephrine

HSCs express 1-adrenoceptors [83], but it is unclear if they also express α-adrenoceptors or which subtypes are expressed. Primary HSCs were found to express the β-adrenoceptors, α-1B, α-1 D, β1, and β2 by RT-PCR analysis. Western blot analysis was used to identify a comparable expression profile. Inhibitor studies provide further evidence that HSCs are both a reservoir and a direct cellular target of NE. They also suggest that the trophic activities of NE require the activation of kinases that enhance cell survival and proliferation [84].

It has recently been demonstrated that hepatocellular carcinoma (HCC) cells, the liver cell line L02, and HSCs (LX2 cells, p-HSC) express adrenergic receptors (ADRs). The mRNA expression of 1 A-ADR was significantly elevated compared to the various expressions of α1B, α1D, β1, β2, and β3-ADR in HSCs. Western blot analysis further supported the pronounced upregulation. HSCs were stimulated by NE administration, which also improved cell proliferation and raised mRNA and protein expression of COL1A1 and α-SMA, two key indicators of HSC activity.

However, the HCC cells did not proliferate more rapidly when exposed to NE at the same concentrations as +HSCs. More significantly, both nonselective and selective α1-ADR antagonists (prazosin and 5-methylurapidi) effectively suppressed NE-dependent HSC activation as demonstrated by decreased expression of α-SMA and Collagen 1 α1, decreased cell proliferation, and enhanced cell death in LX2 cells. Based on these results, NE, a stress hormone, increases HSC activation by activating α1A-ADR signalling [85].

## Neuroimmunomodulation by adrenoblockers in liver cirrhosis

### Advances in the treatments for liver cirrhosis with α and β adrenoblockers

In a mouse model of progenitor cell activation, recent studies demonstrate that inhibition of the sympathetic nervous system, either through α1-adrenergic antagonism with prazosin or chemical sympathectomy with 6-hydroxy dopamine, promotes progenitor cell activation and lessens liver damage [86]. Similar studies on chronically CCL_4_-intoxicated mice revealed that 6-hydroxydopamine and the sympathetic neurotransmitter prazosin prevented fibrosis [85]. Stellate cells may produce and respond to norepinephrine, as demonstrated by other investigators [87]. Recently, acute galactosamine intoxication and acute and chronic CCL_4_ intoxication were investigated. Authors demonstrated that prazosin considerably increased the number of progenitor cells (identified by OV-6) and dramatically decreased the number of hepatic stellate cells (identified by GFAP, desmin, and α-SMA) in acute and chronic rat models. The prazosin-treated animals had less fibrosis than the control animals, supporting the findings. Isolated progenitor and stellate cells both express α-adrenergic receptors. Prazosin is a well-tolerated medication that offers intriguing possibilities for upcoming therapy approaches [85].

In conclusion, the liver has a cell compartment composed of progenitor and hepatic stellate cells with neuroendocrine characteristics. There is growing proof that this cell compartment is influenced by the sympathetic and parasympathetic nervous systems [88]. In hamster models of fibrosis and cirrhosis, it has been concluded that the alpha-adrenoblockers doxazosin and carvedilol reduce type-I collagen by reducing the expression of TGF-β due to receptor blockade. In a similar model, tissue regeneration was also observed [89].

In a hamster liver cirrhosis model, treatment with doxazosin or carvedilol improved histology due to a decrease in collagen fibres in the parenchyma. Carvedilol increased the expression of the cell proliferation markers, α-FP, Ki-67, and c-Myc; however, doxazosin did not show changes in their expression, so these adrenoblockers are proposed as treatment in the regression of chronic heart diseases. Unfortunately, the drugs showed minimal alterations in hepatocyte morphology [90].

Curcumin, an antioxidant and anti-inflammatory compound, has been shown to reverse liver cirrhosis. Investigations in the potential modulation of Nrf-2 and NF-κB in hamster models of carbon tetrachloride (CCl_4_)-induced cirrhosis showed that the toxicity produced by the metabolism of these antagonists decreases. Therapy with carvedilol and the addition of doxazosin to curcumin raised the Nrf-2/NF-κB mRNA ratio and its protein expression in inflammatory liver cells, presumably as an additional hepatoprotective mechanism [91].

According to authors, carvedilol’s antioxidant and anti-inflammatory properties can lessen hepatocyte damage, stop hepatic stellate cell activation, and reduce collagen production. The results of a histological investigation confirmed the antifibrotic action of carvedilol by demonstrating how co-treatment with carvedilol reduced the histopathological changes caused by CCl_4_ [92]. After CCl_4_ intoxication, fibrotic lesions were confirmed by Masson’s trichrome stain, while carvedilol-treated CCl_4_-intoxicated rats simultaneously showed similar normal levels of TGF-β1, hydroxyproline, and minimal collagen deposition. Serum albumin and total protein measurements were used to demonstrate the hepatic synthesis capability and the antifibrotic effects of carvedilol. It was reported that both experienced significant depletion from persistent CCl_4_ intoxication, but intoxicated rats treated with concomitant carvedilol showed a return to normal levels [92].

**Figure 3.**
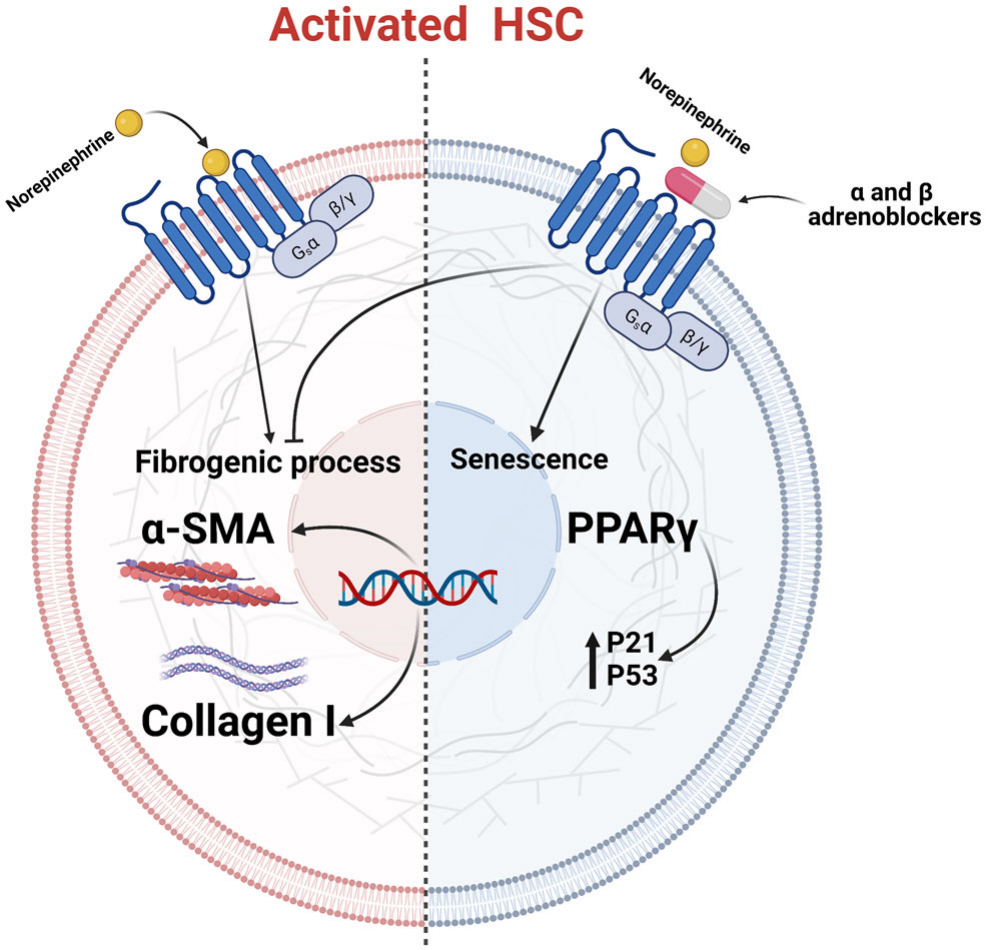
PPAR-γ expression is elevated in quiescent HSC and is overexpressed in active cells once they enter senescence due to adrenergic receptor blockade.

Reported research [93] demonstrated that the α1-adrenoblocker doxazosin decreased the fibrogenic activity of activated HSCs, which is connected to the induction of cellular senescence *via* α1 AR antagonism, suggesting that α1-AR is a potential treatment for liver fibrosis. The adrenoblocker downregulated collagen 1 and ACTA2 and increased the expression of PPAR-γ in the presence and absence of TGF-β, confirming that doxazosin delays stellate cell activation (Figure 3). The peroxisome proliferators control the activation of the nuclear hormone receptor superfamily that includes the PPARs. PPAR-γ is activated following ligand binding and joins with retinoids X receptor (RXR) to form a heterodimer. High levels of PPAR-γ expression are found in HSCs that are quiescent and inhibited during liver cirrhosis. According to studies, activating PPAR-γ inhibits HSC activation and decreases collagen deposition during liver injury [94].

Curcumin inhibited TGF-β signalling by increasing the expression of PPAR-γ, which in turn blocked connective tissue growth factor and pro-collagen I from being expressed in activated HSCs in a dose-dependent manner [95]. Thiazolidinedione (TZD), a PPAR-γ agonist, keeps HSCs quiescence in an *in vivo* model of hepatic fibrosis by controlling adipogenicity transcription factors, which also prevents HSCs from becoming activated shown by α-SMA immunostaining and producing collagen I with Sirius Red staining [96]. However, GW570 nonthiozolidinedione PPAR agonist inhibited collagen I α1 and α-SMA mRNA and protein expression on isolated stellate cells in *in vivo* model of hepatic fibrosis because physical interaction between PPAR-γ and JunD in stellate cells suppresses AP-1 activity, which prevents their activation [97].

A sustained senescence-associated secretory phenotype can negatively affect tissues and organs because the proinflammatory profile of senescent cells can increase epithelial cell proliferation and tumorigenesis, even though senescence induction is a promising therapeutic strategy to reverse hepatic fibrogenesis [86].

Studies demonstrate the use of α and β adrenoblockers in *in vitro* and *in vivo* preclinical treatments for different chronic liver diseases, such as fibrosis and cirrhosis, that generally target liver stellate cells (Table 1).

**Table 1.** α and β adrenoblockers used in preclinical studies for chronic liver diseases.

Adrenergic Antagonists	Chemical structure	Adrenergic receptor target	Target cell or tissue to control hepatic fibrosis	Research Status	Reference
Prazosin	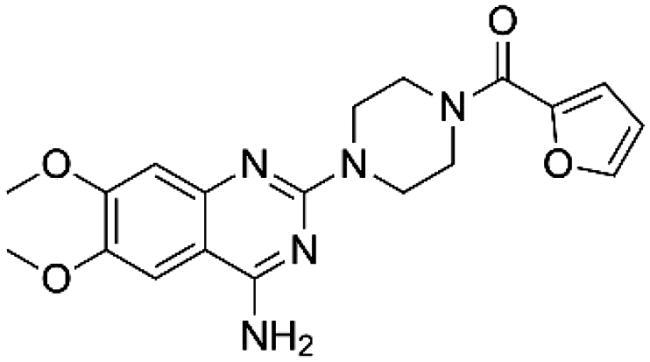	Alpha 1 non-selective	Hepatic Stellar Cell	Preclinical study	Dubuisson et al. [98] Sancho-Bru et al. [81] Sigala et al. [99] Svegliati-Baroni et al. [100] Oben et al. [101] Oben et al. [81] Lin et al. [85]
Doxazosin	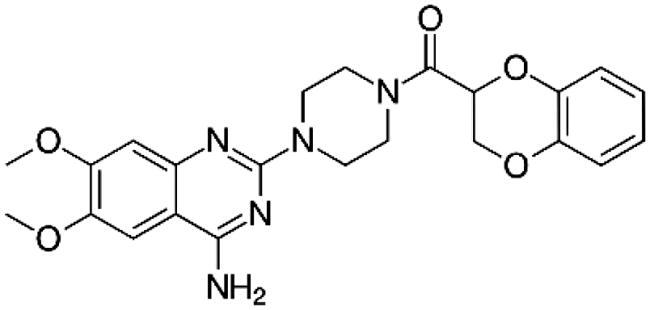	Alpha 1 non-selective	Hepatic Stellar Cell	Preclinical study	Muñoz-Ortega et al. [89] Cervantes-García et al. [102] Serna-Salas et al. [90, 93] Macias-Pérez et al. [91] Xiu et al. [103]
5-Methylurapidil	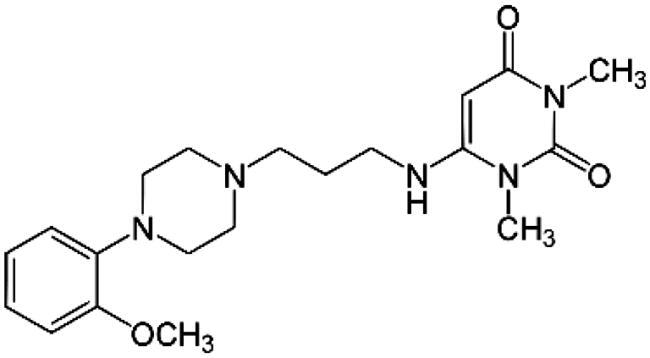	Alpha 1 selective to subtype A	Hepatic Stellar Cell	LX-2 cell line *in vitro* study	Lin et al. [85]
Propranolol	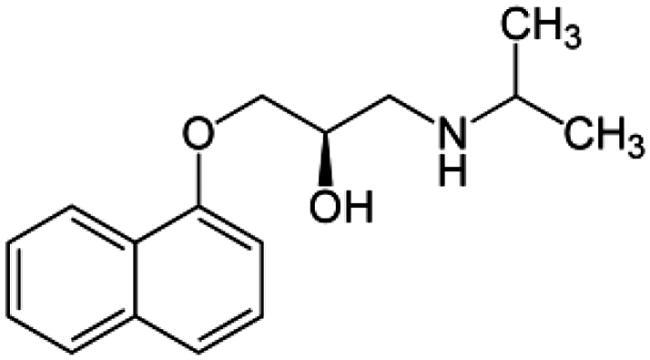	Non-selective beta subtypes beta 1 and 2	Hepatic Stellar Cell and Portal Circulation	Preclinical study for portal hypertension	Ding et al. [104] Mende et al. [105] Strack et al. [106] D’Amico et al. [107] Abdel-Kawy et al. [108] McKee et al. [83] Oben et al. [87] Oben et al. [109] Schepke et al. [110] Hobolth et al. [111] Aguilar-Olivos et al. [112] Suna et al. [113] Chun-Chen et al. [114]
Carvedilol	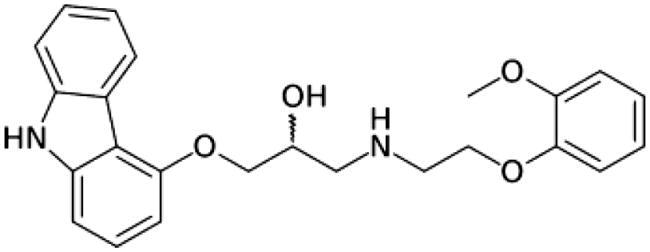	Alpha 1 non-selective and beta non-selective subtypes beta 1 and 2	Hepatic Stellar Cell and Portal Circulation	Preclinical study for portal hypertension	Hamdy et al. [92] Muñoz-Ortega et al. [89] El-Demerdash et al. [115] Meng et al. [116] Serna-Salas et al. [90] Wu et al. [117] Ling et al. [118] Bosch [119] Aguilar-Olivos et al. [112] Hobolth et al. [111] Abdel-Kawy et al. [108]
Atenolol	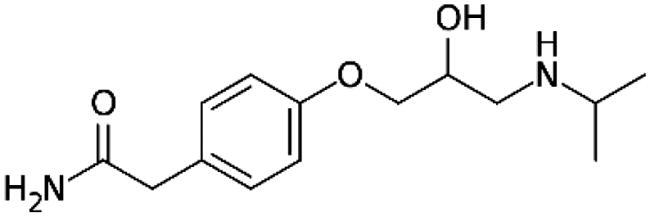	Selective beta subtype Beta 1	Portal Circulation	Preclinical study for portal hypertension	Mikheeva et al. [120]

## Conclusions and future directions

**Figure 4.**
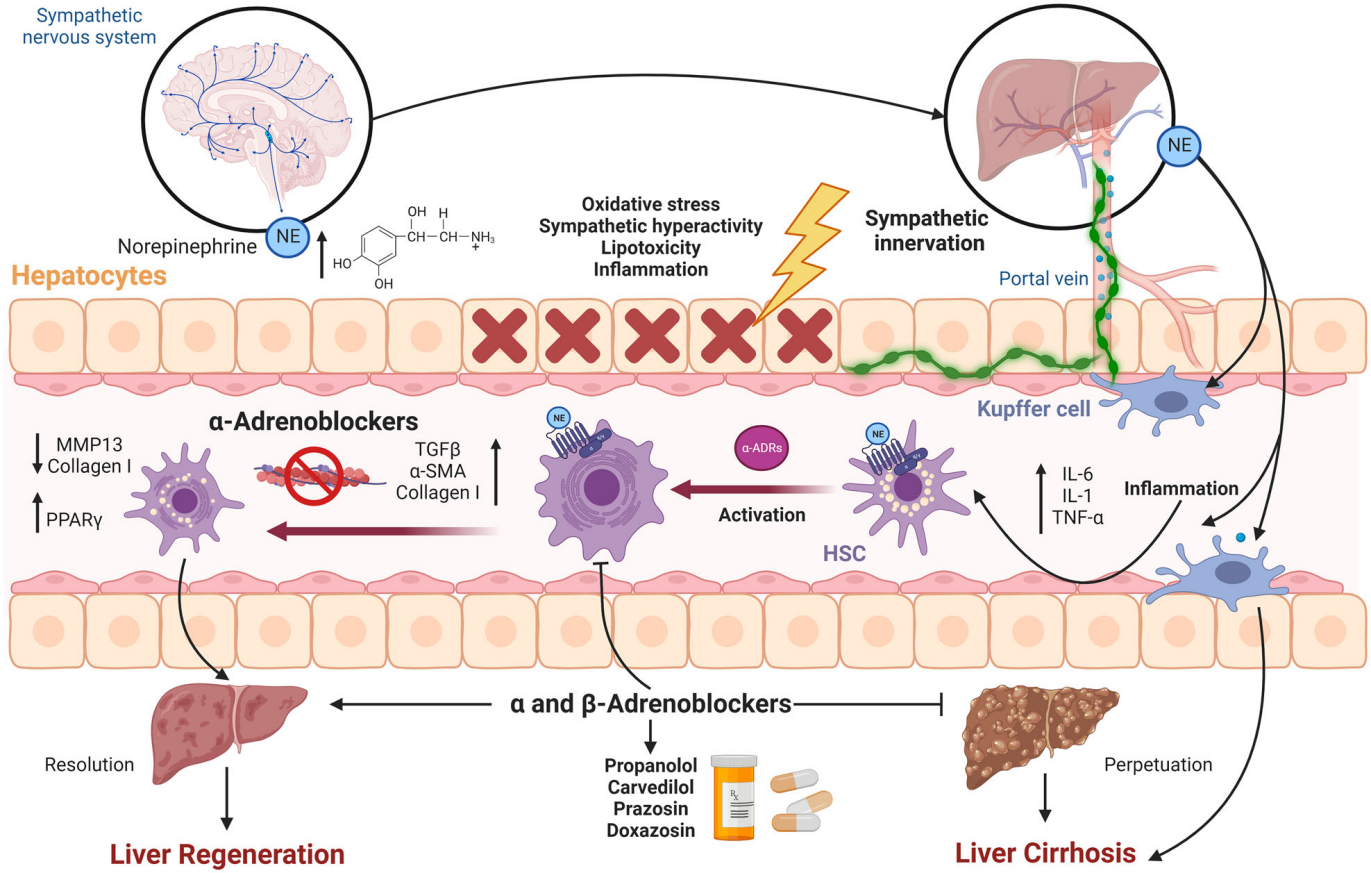
The sympathetic nervous system and the immune system communicate through adrenergic neurotransmitters and cytokines, promoting the activation of HSC and, therefore, fibrosis; however, alpha adrenoblockers can be used as treatments for hepatic cirrhosis.

Possible treatments for hepatic fibrosis and cirrhosis might depend on neurotransmitter and hepatic nervous system regulation. By enhancing HSC activation and proliferation along with increasing cytokine signaling, SNS stimulation aids in promoting hepatic fibrosis. Culturing normal HSCs with α or β adrenoceptor antagonists demonstrated that HSCs express essential enzymes for catecholamine biosynthesis and produce NE and other catecholamines. HSCs use these compounds to autoregulate their growth. By targeting the interruption of catecholamine signaling in hepatic stellate cells, a prospective therapeutic strategy to control the fibrogenic response to liver injury may be proposed by understanding the mechanisms that mediate the profibrogenic activities of catecholamines in the liver. Figure 4 illustrates strategies to reduce hepatic stellate cell activation and, thus, fibrosis based on experimentation with α and β adrenoblockers.

## Data Availability

The data supporting the findings of this study are available on request from the corresponding author (MHMO).

## References

[1] Felten DL, Livnat S, Felten SY, et al. Sympathetic innervation of lymph nodes in mice. Brain Res Bull. 1984;13(6):693–699. 6532515 10.1016/0361-9230(84)90230-2

[2] Tracey KJ. Reflex control of immunity. Nat Rev Immunol. 2009;9(6):418–428. 19461672 10.1038/nri2566 PMC4535331

[3] Gidron Y, Kupper N, Kwaijtaal M, et al. Vagus-Brain communication in Atherosclerosis-Related inflammation: a neuroimmunomodulation perspective of CAD. Atherosclerosis. 2007;195(2):e1–e9. 10.1016/j.atherosclerosis.2006.10.009 17101139

[4] Gidron Y, Perry H, Glennie M. Does the vagus nerve inform the brain about preclinical tumours and modulate them? Lancet Oncol. 2005;6(4):245–248. 15811620 10.1016/S1470-2045(05)70096-6

[5] Davidson RJ, Coe CC, Dolski I, et al. Individual differences in prefrontal activation asymmetry predict natural killer cell activity at rest and in response to challenge. Brain Behav Immun. 1999;13(2):93–108. 10373275 10.1006/brbi.1999.0557

[6] Meador KJ, Loring DW, Ray PG, et al. Role of cerebral lateralization in control of immune processes in humans. Ann Neurol. 2004;55(6):840–844. 15174018 10.1002/ana.20105

[7] Lewis RS, Weekes NY, Wang TH. The effect of a naturalistic stressor on frontal EEG asymmetry, stress, and health. Biol Psychol. 2007;75(3):239–247. 17512106 10.1016/j.biopsycho.2007.03.004

[8] Gidron Y, Hall P, Wesnes KA, et al. Does a neuropsychological index of hemispheric lateralization predict onset of upper respiratory tract infectious symptoms? Br J Health Psychol. 2010;15(Pt 3):469–477. 19769796 10.1348/135910709X471391

[9] Wehrwein EA, Orer HS, Barman SM. Overview of the anatomy. Compr Physiol. 2016;6(3):1239–1278. 27347892 10.1002/cphy.c150037

[10] Shibasaki M, Crandall CG. Mechanisms and controllers of eccrine sweating in humans. Front Biosci (Schol Ed). 2010;2(2):685–696. 20036977 10.2741/s94 PMC2866164

[11] Strosberg AD. Structure, function, and regulation of adrenergic receptors. Protein Sci. 1993;2(8):1198–1209. 8401205 10.1002/pro.5560020802 PMC2142449

[12] Elenkov IJ, Wilder RL, Chrousos GP, et al. The sympathetic nerve–an integrative interface between two supersystems: the brain and the immune system. Pharmacol Rev. 2000;52(4):595–638. 11121511

[13] Zurier RB, Weissmann G, Hoffstein S, et al. Mechanisms of lysosomal enzyme release from human leukocytes II. EFFECTS oF CAMP aND CGMP, AUTONOMIC AGONISTS, and AGENTS WHICH AFFECT MICROTUBULE FUNCTION. J Clin Invest. 1974;53(1):297–309. 4357615 10.1172/JCI107550 PMC301465

[14] Nielson CP. Beta-Adrenergic modulation of the polymorphonuclear leukocyte respiratory burst is dependent upon the mechanism of cell activation. J Immunol. 1987;139(7):2392–2397. 2821113

[15] Picklo MJ. Methods of sympathetic degeneration and alteration. J Auton Nerv Syst. 1997;62(3):111–125. 9051618 10.1016/s0165-1838(96)00121-x

[16] Ackerman KD, Madden KS, Livnat S, et al. Neonatal sympathetic denervation alters the development of in vitro spleen cell proliferation and differentiation. Brain Behav Immun. 1991;5(3):235–261. 1954402 10.1016/0889-1591(91)90021-2

[17] Neuhuber WL, Tiegs G. Innervation of immune cells: evidence for neuroimmunomodulation in the liver. Anat Rec A Discov Mol Cell Evol Biol. 2004;280(1):884–892. 15382013 10.1002/ar.a.20093

[18] Forssmann WG, Ito S. Hepatocyte innervation in primates. J Cell Biol. 1977;74(1):299–313. 406265 10.1083/jcb.74.1.299 PMC2109862

[19] Jensen KJ, Alpini G, Glaser S. Hepatic nervous system and neurobiology of the liver. Compr Physiol. 2013;3(2):655–665. 23720325 10.1002/cphy.c120018 PMC3733049

[20] Gardemann A, Jungermann K. Control of glucose balance in the perfused rat liver by the parasympathetic innervation. Biol Chem Hoppe Seyler. 1986;367(7):559–566. 3530281 10.1515/bchm3.1986.367.2.559

[21] Cassiman D, Libbrecht L, Sinelli N, et al. The vagal nerve stimulates activation of the hepatic progenitor cell compartment via muscarinic acetylcholine receptor type 3. Am J Pathol. 2002;161(2):521–530. 12163377 10.1016/S0002-9440(10)64208-3 PMC1850744

[22] Tracey KJ. The inflammatory reflex. Nature. 2002;420(6917):853–859. 12490958 10.1038/nature01321

[23] Anthony PP, Ishak KG, Nayak NC, et al. The morphology of cirrhosis: definition, nomenclature, and classification. Bull World Health Organ. 1977;55(4):521–540. 304393 PMC2366674

[24] Ellis EL, Mann DA. Clinical evidence for the regression of liver fibrosis. J Hepatol. 2012;56(5):1171–1180. 22245903 10.1016/j.jhep.2011.09.024

[25] Oakley F, Meso M, Iredale JP, et al. Inhibition of inhibitor of KappaB kinases stimulates hepatic stellate cell apoptosis and accelerated recovery from rat liver fibrosis. Gastroenterology. 2005;128(1):108–120. 15633128 10.1053/j.gastro.2004.10.003

[26] Friedman SL. Evolving challenges in hepatic fibrosis. Nat Rev Gastroenterol Hepatol. 2010;7(8):425–436. 20585339 10.1038/nrgastro.2010.97

[27] Grappone C, Pinzani M, Parola M, et al. Expression of Platelet-Derived growth factor in newly formed cholangiocytes during experimental biliary fibrosis in rats. J Hepatol. 1999;31(1):100–109. 10424289 10.1016/s0168-8278(99)80169-x

[28] Ramm GA, Nair VG, Bridle KR, et al. Contribution of hepatic parenchymal and nonparenchymal cells to hepatic fibrogenesis in biliary atresia. Am J Pathol. 1998;153(2):527–535. 9708812 10.1016/S0002-9440(10)65595-2 PMC1852970

[29] Milani S, Herbst H, Schuppan D, et al. Transforming growth factors beta 1 and beta 2 are differentially expressed in fibrotic liver disease. Am J Pathol. 1991;139(6):1221–1229. 1750499 PMC1886459

[30] Sedlaczek N, Jia JD, Bauer M, et al. Proliferating bile duct epithelial cells are a major source of connective tissue growth factor in rat biliary fibrosis. Am J Pathol. 2001;158(4):1239–1244. 11290541 10.1016/S0002-9440(10)64074-6 PMC1891909

[31] Lo RC, Ng, IO. Hepatic Progenitor Cells: Their Role and Functional Significance in the New Classification of Primary Liver Cancers. Liver Cancer. 2013;2(2):84. 10.1159/000343844 PMC3740719 24159600

[32] Bhunchet E, Fujieda K. Capillarization and venularization of hepatic sinusoids in porcine Serum-Induced rat liver fibrosis: a mechanism to maintain liver blood flow. Hepatology. 1993;18(6):1450–1458. 7694897

[33] Mori T, Okanoue T, Sawa Y, et al. Defenestration of the sinusoidal endothelial cell in a rat model of cirrhosis. Hepatology. 1993;17(5):891–897. 8491454

[34] Yokomori H, Oda M, Yoshimura K, et al. Recent advances in liver sinusoidal endothelial ultrastructure and fine structure immunocytochemistry. Micron. 2012;43(2-3):129–134. 21906955 10.1016/j.micron.2011.08.002

[35] Marvie P, Lisbonne M, L'helgoualc’h A, et al. Interleukin-33 overexpression is associated with liver fibrosis in mice and humans. J Cell Mol Med. 2010;14(6b):1726–1739. 19508382 10.1111/j.1582-4934.2009.00801.x PMC3829034

[36] DeLeve LD, Wang X, Guo Y. Sinusoidal endothelial cells prevent rat stellate cell activation and promote reversion to quiescence. Hepatology. 2008;48(3):920–930. 18613151 10.1002/hep.22351 PMC2695448

[37] Hamazaki K, Sato S, Yunoki M, et al. Experimental medicine kupffer cell function in chronic liver injury and after partial hepatectomy. Res Exp Med. 1994;194(4):237–246. 10.1007/BF02576385 7800933

[38] López-Navarrete G, Ramos-Martínez E, Suárez-Álvarez K, et al. Th2-Associated alternative kupffer cell activation promotes liver fibrosis without inducing local inflammation. Int J Biol Sci. 2011;7(9):1273–1286. 22110380 10.7150/ijbs.7.1273 PMC3221364

[39] Friedman SL, Arthur, MJ Activation of cultured rat hepatic lipocytes by kupffer cell conditioned medium. Direct enhancement of matrix synthesis and stimulation of cell proliferation via induction of Platelet-Derived growth factor receptors. J Clin Invest. 1989;84(6):1780–1785. 2556445 10.1172/JCI114362 PMC304055

[40] Matsuoka M, Tsukamoto H. Stimulation of hepatic lipocyte collagen production by kupffer Cell-Derived transforming growth factor β: implication for a pathogenetic role in alcoholic liver fibrogenesis. Hepatology. 1990;11(4):599–605. 2328954 10.1002/hep.1840110412

[41] Benyon RC, Hovell CJ, Da Gaça M, et al. Progelatinase a is produced and activated by rat hepatic stellate cells and promotes their proliferation. Hepatology. 1999;30(4):977–986. 10498650 10.1002/hep.510300431

[42] Canbay A, Feldstein AE, Higuchi H, et al. Kupffer cell engulfment of apoptotic bodies stimulates death ligand and cytokine expression. Hepatology. 2003;38(5):1188–1198. 14578857 10.1053/jhep.2003.50472

[43] Steib CJ, Gerbes AL, Bystron M, et al. Kupffer cell activation in normal and fibrotic livers increases portal pressure via thromboxane A(2). J Hepatol. 2007;47(2):228–238. 17573142 10.1016/j.jhep.2007.03.019

[44] Schattenberg JM, Nagel M, Kim YO, et al. Increased hepatic fibrosis and JNK2-Dependent liver injury in mice exhibiting Hepatocyte-Specific deletion of CFLIP. Am J Physiol Gastrointest Liver Physiol. 2012;303(4):G498–G4506. . 22700824 10.1152/ajpgi.00525.2011

[45] Chen L, Yang Z, Qiu F. Studies on hepatocyte apoptosis, proliferation and oncogene c-Fos expression in carbon Tetrachloride-Induced cirrhotic rat liver. J Tongji Med Univ. 1999;19(1):53–55. 12840877 10.1007/BF02895597

[46] del Carmen Garcíade León M, Montfort I, Tello Montes E, et al. Hepatocyte production of modulators of extracellular liver matrix in normal and cirrhotic rat liver. Exp Mol Pathol. 2006;80(1):97–108. 16332368 10.1016/j.yexmp.2005.03.008

[47] Jeong W I, Do SH, Yun HS, et al. Hypoxia potentiates transforming growth Factor-Beta expression of hepatocyte during the cirrhotic condition in rat liver. Liver Int. 2004;24(6):658–668. 15566519 10.1111/j.1478-3231.2004.0961.x

[48] Wiemann SU, Satyanarayana A, Tsahuridu M, et al. Hepatocyte telomere shortening and senescence are general markers of human liver cirrhosis. Faseb J. 2002;16(9):935–942. 12087054 10.1096/fj.01-0977com

[49] Martin I. V, Borkham-Kamphorst E, Zok S, et al. Platelet-Derived growth factor (PDGF)-C neutralization reveals differential roles of PDGF receptors in liver and kidney fibrosis. Am J Pathol. 2013;182(1):107–117. 23141925 10.1016/j.ajpath.2012.09.006

[50] Thieringer F, Maass T, Czochra P, et al. Spontaneous hepatic fibrosis in transgenic mice overexpressing PDGF-A. Gene. 2008;423(1):23–28. 18598744 10.1016/j.gene.2008.05.022

[51] Borkham-Kamphorst E, Herrmann J, Stoll D, et al. Dominant-Negative soluble PDGF-β receptor inhibits hepatic stellate cell activation and attenuates liver fibrosis. Lab Invest. 2004;84(6):766–777. 15077122 10.1038/labinvest.3700094

[52] Czochra P, Klopcic B, Meyer E, et al. Liver fibrosis induced by hepatic overexpression of PDGF-B in transgenic mice. J Hepatol. 2006;45(3):419–428. 16842882 10.1016/j.jhep.2006.04.010

[53] Ogawa S, Ochi T, Shimada H, et al. Anti-PDGF-B monoclonal antibody reduces liver fibrosis development. Hepatol Res. 2010;40(11):1128–1141. 20880061 10.1111/j.1872-034X.2010.00718.x

[54] Borkham-Kamphorst E, van Roeyen CRC, Ostendorf T, et al. Pro-Fibrogenic potential of PDGF-D in liver fibrosis. J Hepatol. 2007;46(6):1064–1074. 17397961 10.1016/j.jhep.2007.01.029

[55] Yokoyama H, Masaki T, Inoue I, et al. Histological and biochemical evaluation of transforming growth factor-β activation and its clinical significance in patients with chronic liver disease. Heliyon. 2019;5(2):e01231. 30815603 10.1016/j.heliyon.2019.e01231 PMC6378908

[56] Cui Q, Wang Z, Jiang D, et al. HGF inhibits TGF-Β1-Induced myofibroblast differentiation and ECM deposition via MMP-2 in achilles tendon in rat. Eur J Appl Physiol. 2011;111(7):1457–1463. 21165643 10.1007/s00421-010-1764-4

[57] Castilla A, Prieto J, Fausto N. Transforming growth factors Β1 and α in chronic liver disease. N Engl J Med. 2010;324(14):933–940. 10.1056/NEJM199104043241401 1900574

[58] Crespo J, Rivero M, Fábrega E, et al. Plasma leptin and TNF-Alpha levels in chronic hepatitis C patients and their relationship to hepatic fibrosis. Dig Dis Sci. 2002;47(7):1604–1610. 12141823 10.1023/a:1015835606718

[59] Saile B, Matthes N, el Armouche H, et al. The bcl, NFκB and P53/P21WAF1 systems are involved in spontaneous apoptosis and in the anti-Apoptotic effect of TGF-β or TNF-α on activated hepatic stellate cells. Eur J Cell Biol. 2001;80(8):554–561. 11561906 10.1078/0171-9335-00182

[60] Taimr P, Higuchi H, Kocova E, et al. Activated stellate cells express the TRAIL receptor-2/death receptor-5 and undergo TRAIL-Mediated apoptosis. Hepatology. 2003;37(1):87–95. 12500193 10.1053/jhep.2003.50002

[61] Varela-Rey M, Fontán-Gabás L, Blanco P, et al. Glutathione depletion is involved in the inhibition of procollagen Α1(I) MRNA levels caused by TNF-α on hepatic stellate cells. Cytokine. 2007;37(3):212–217. 17485223 10.1016/j.cyto.2007.03.013

[62] Poynard T, Massard J, Rudler M, et al. Impact of Interferon-Alpha treatment on liver fibrosis in patients with chronic hepatitis B: an overview of published trials. Gastroenterol Clin Biol. 2009;33(10-11):916–922. 19640664 10.1016/j.gcb.2009.06.006

[63] Ogawa T, Kawada N, Ikeda K. Effect of natural interferon α on proliferation and apoptosis of hepatic stellate cells. Hepatol Int. 2009;3(3):497–503. 19669254 10.1007/s12072-009-9129-y PMC2748375

[64] Rao HY, Wei L, Wang JH, et al. Inhibitory effect of human Interferon-Beta-1a on activated rat and human hepatic stellate cells. J Gastroenterol Hepatol. 2010;25(11):1777–1784. 21039841 10.1111/j.1440-1746.2010.06264.x

[65] Baroni GS, D'Ambrosio L, Curto P, et al. Interferon gamma decreases hepatic stellate cell activation and extracellular matrix deposition in rat liver fibrosis. Hepatology. 1996;23(5):1189–1199. 8621153 10.1002/hep.510230538

[66] Zhou WC, Zhang QB, Qiao L. Pathogenesis of liver cirrhosis. World J Gastroenterol. 2014;20(23):7312–7324. 24966602 10.3748/wjg.v20.i23.7312 PMC4064077

[67] Gieling RG, Wallace K, Han YP. Interleukin-1 participates in the progression from liver injury to fibrosis. Am J Physiol Gastrointest Liver Physiol. 2009;296(6):G1324–G1331. 19342509 10.1152/ajpgi.90564.2008 PMC2697947

[68] Mancini R, Benedetti A, Jezequel A-M, et al. An interleukin-1 receptor antagonist decreases fibrosis induced by dimethylnitrosamine in rat liver. Virchows Arch. 1994;424(1):25–31. 7981900 10.1007/BF00197389

[69] Petrasek J, Bala S, Csak T, et al. IL-1 receptor antagonist ameliorates Inflammasome-Dependent alcoholic steatohepatitis in mice. J Clin Invest. 2012;122(10):3476–3489. 22945633 10.1172/JCI60777 PMC3461900

[70] Du WJ, Zhen JH, Zeng ZQ, et al. Expression of interleukin-17 associated with disease progression and liver fibrosis with hepatitis B virus infection: IL-17 in HBV infection. Diagn Pathol. 2013;8(1):40. 23448394 10.1186/1746-1596-8-40 PMC3598543

[71] Meng F, Wang K, Aoyama T, et al. Interleukin-17 signaling in inflammatory, kupffer cells, and hepatic stellate cells exacerbates liver fibrosis in mice. Gastroenterology. 2012;143(3):765–776.e3. 22687286 10.1053/j.gastro.2012.05.049 PMC3635475

[72] Chou WY, Lu CN, Lee TH, et al. Electroporative interleukin-10 gene transfer ameliorates carbon Tetrachloride-Induced murine liver fibrosis by MMP and TIMP modulation. Acta Pharmacol Sin. 2006;27(4):469–476. 16539848 10.1111/j.1745-7254.2006.00304.x

[73] Zhang L-J, Zheng W-D, Chen Y-X, et al. Antifibrotic effects of interleukin-10 on experimental hepatic fibrosis. Hepatogastroenterology. 2007;54(79):2092–2098. 18251166

[74] Huang YH, Shi MN, Zhen W, et al. Therapeutic effect of interleukin-10 on CCl_4_-Induced hepatic fibrosis in rats. World J Gastroenterol. 2006;12(9):1386–1391. 16552806 10.3748/wjg.v12.i9.1386 PMC4124315

[75] Kong X, Feng D, Wang H, et al. Interleukin-22 induces hepatic stellate cell senescence and restricts liver fibrosis in mice. Hepatology. 2012;56(3):1150–1159. 22473749 10.1002/hep.25744 PMC3394879

[76] Kovalovich K, Deangelis RA, Li W, et al. Increased Toxin-Induced liver injury and fibrosis in interleukin-6-Deficient mice. Hepatology. 2000;31(1):149–159. 10613740 10.1002/hep.510310123

[77] Nasir GA, Mohsin S, Khan M, et al. Mesenchymal stem cells and interleukin-6 attenuate liver fibrosis in mice. J Transl Med. 2013;11(1):78. 23531302 10.1186/1479-5876-11-78 PMC3636128

[78] Elenkov IJ, Chrousos GP. Stress hormones, Th1/Th2 patterns, pro/anti-Inflammatory cytokines and susceptibility to disease. Trends Endocrinol Metab. 1999;10(9):359–368. 10511695 10.1016/s1043-2760(99)00188-5

[79] Felsner P, Hofer D, Rinner I, et al. Adrenergic suppression of peripheral blood T cell reactivity in the rat is due to activation of peripheral Α2-Receptors. J Neuroimmunol. 1995;57(1-2):27–34. 7706438 10.1016/0165-5728(94)00158-k

[80] Felsner P, Hofer D, Rinner I, et al. Continuous in vivo treatment with catecholamines suppresses in vitro reactivity of rat peripheral blood T-Lymphocytes via Alpha-Mediated mechanisms. J Neuroimmunol. 1992;37(1–2):47–57. 1372330 10.1016/0165-5728(92)90154-d

[81] Oben JA, Diehl AM. Sympathetic nervous system regulation of liver repair. Anat. Rec. September 2004;280A(1)pp :874–883. 10.1002/ar.a.20081 15382023

[82] Sigala B, McKee C, Soeda J, et al. Sympathetic nervous system catecholamines and neuropeptide Y neurotransmitters are upregulated in human NAFLD and modulate the fibrogenic function of hepatic stellate cells. PLoS One. 2013;8(9):e72928. 24019886 10.1371/journal.pone.0072928 PMC3760858

[83] Athari A, Hänecke K, Jungermann K. Prostaglandin F2α and D2 release from primary ito cell cultures after stimulation with noradrenaline and ATP but not adenosine. Hepatology. 1994;20(1 Pt 1):142–148. 8020883 10.1016/0270-9139(94)90146-5

[84] Oben JA, Roskams T, Yang S, et al. Hepatic fibrogenesis requires sympathetic neurotransmitters. Gut. 2004;53(3):438–445. 14960531 10.1136/gut.2003.026658 PMC1773985

[85] Lin XH, Liu HH, Hsu SJ, et al. Norepinephrine-Stimulated HSCs secrete SFRP1 to promote HCC progression following chronic stress via augmentation of a Wnt16B/β-Catenin positive feedback loop. J Exp Clin Cancer Res. 2020;39(1):64. 32293507 10.1186/s13046-020-01568-0 PMC7158101

[86] Oben JA, Roskams T, Yang S, et al. Sympathetic nervous system inhibition increases hepatic progenitors and reduces liver injury. Hepatology. 2003;38(3):664–673. 12939593 10.1053/jhep.2003.50371

[87] Dubuisson L, Desmoulière A, Decourt B, et al. Inhibition of rat liver fibrogenesis through noradrenergic antagonism. Hepatology. 2002;35(2):325–331. 11826405 10.1053/jhep.2002.31166

[88] Roskams T, Cassiman D, de Vos R, et al. Neuroregulation of the neuroendocrine compartment of the liver. Anat Rec A Discov Mol Cell Evol Biol. 2004;280 *A*(1):910–923. 15382010 10.1002/ar.a.20096

[89] Muñoz-Ortega MH, Llamas-Ramírez RW, Romero-Delgadillo NI, et al. Doxazosin treatment attenuates carbon Tetrachloride-Induced liver fibrosis in hamsters through a decrease in transforming growth factor β secretion. Gut Liver. 2016;10(1):101–108. 26573293 10.5009/gnl14459 PMC4694741

[90] Serna-Salas SA, Navarro-González YD, Martínez-Hernández SL, et al. Doxazosin and carvedilol treatment improves hepatic regeneration in a hamster model of cirrhosis. Biomed Res Int. 2018;2018:4706976. 30643808 10.1155/2018/4706976 PMC6311259

[91] Macías-Pérez JR, Vázquez-López BJ, Muñoz-Ortega MH, et al. Curcumin and α/β-Adrenergic antagonists cotreatment reverse liver cirrhosis in hamsters: participation of nrf-2 and NF- κ B. J Immunol Res. 2019;2019:3019794. 31183386 10.1155/2019/3019794 PMC6515016

[92] Hamdy N, El-Demerdash E. New therapeutic aspect for carvedilol: antifibrotic effects of carvedilol in chronic carbon Tetrachloride-Induced liver damage. Toxicol Appl Pharmacol. 2012;261(3):292–299. 22543095 10.1016/j.taap.2012.04.012

[93] Serna-Salas SA, Arroyave-Ospina JC, Zhang M, et al. α-1 adrenergic receptor antagonist doxazosin reverses hepatic stellate cells activation via induction of senescence. Mech Ageing Dev. 2022;201:111617. 34958827 10.1016/j.mad.2021.111617

[94] Wu L, Guo C, Wu J. Therapeutic potential of PPARγ natural agonists in liver diseases. J Cell Mol Med. 2020;24(5):2736–2748. 32031298 10.1111/jcmm.15028 PMC7077554

[95] Zheng S, Chen A. Curcumin suppresses the expression of extracellular matrix genes in activated hepatic stellate cells by inhibiting gene expression of connective tissue growth factor. Am J Physiol Gastrointest Liver Physiol. 2006;290(5):G883–G893. . 16306131 10.1152/ajpgi.00450.2005

[96] Alatas FS, Matsuura T, Pudjiadi AH, et al. Peroxisome Proliferator-Activated receptor gamma agonist attenuates liver fibrosis by several fibrogenic pathways in an animal model of cholestatic fibrosis. Pediatr Gastroenterol Hepatol Nutr. 2020;23(4):346–355. 32704495 10.5223/pghn.2020.23.4.346 PMC7354870

[97] Yang L, Stimpson SA, Chen L, et al. Effectiveness of the PPARγ agonist, GW570, in liver fibrosis. Inflamm Res. 2010;59(12):1061–1071. 20585829 10.1007/s00011-010-0226-0

[98] Laberge RM, Awad P, Campisi J, et al. Epithelial-Mesenchymal transition induced by senescent fibroblasts. Cancer Microenvironment. 2012;5(1):39–44. 21706180 10.1007/s12307-011-0069-4 PMC3343197

[99] Sancho-Bru P, Bataller R, Colmenero J, et al. Norepinephrine induces calcium spikes and proinflammatory actions in human hepatic stellate cells. Am J Physiol Gastrointest Liver Physiol. 2006;291(5):877–884. 10.1152/ajpgi.00537.2005 16782692

[100] Svegliati-Baroni G, de Minicis S, Marzioni M. Hepatic fibrogenesis in response to chronic liver injury: novel insights on the role of cell-to-Cell interaction and transition. Liver Int. 2008;28(8):1052–1064. 18783548 10.1111/j.1478-3231.2008.01825.x

[101] Oben JA, Yang S, Lin H, et al. Norepinephrine and neuropeptide Y promote proliferation and collagen gene expression of hepatic myofibroblastic stellate cells. Biochem Biophys Res Commun. 2003;302(4):685–690. 12646223 10.1016/s0006-291x(03)00232-8

[102] Cervantes-Garcia D, Cuellar-Juarez AG, Borrego-Soto G, et al. Adenoviral-bone morphogenetic protein-7 and/or doxazosin therapies promote the reversion of fibrosis/cirrhosis in a cirrhotic hamster model. Mol Med Rep. 2017;16(6):9431–9440. 29039539 10.3892/mmr.2017.7785 PMC5780000

[103] Xiu AY, Ding Q, Li Z, et al. Doxazosin attenuates liver fibrosis by inhibiting autophagy in hepatic stellate cells via activation of the PI3K/akt/MTOR signaling pathway. Drug Des Devel Ther. 2021;15:3643–3659. 10.2147/DDDT.S317701 PMC8387324 34456560

[104] Ding Q, Li Z, Liu B, et al. Propranolol prevents liver cirrhosis by inhibiting hepatic stellate cell activation mediated by the PDGFR/akt pathway. Hum Pathol. 2018;76:37–46. 29514109 10.1016/j.humpath.2018.02.018

[105] Mende S, Schulte S, Strack I, et al. Telmisartan plus propranolol improves liver fibrosis and bile duct proliferation in the PSC-Like Abcb4−/− mouse model. Dig Dis Sci. 2013;58(5):1271–1281. 23247798 10.1007/s10620-012-2499-3

[106] Strack I, Schulte S, Varnholt H, et al. β-Adrenoceptor blockade in sclerosing cholangitis of Mdr2 knockout mice: antifibrotic effects in a model of nonsinusoidal fibrosis. Lab Invest. 2011;91(2):252–261. 20921947 10.1038/labinvest.2010.162

[107] D'Amico M, Mejías M, García-Pras E, et al. Effects of the combined administration of propranolol plus sorafenib on portal hypertension in cirrhotic rats. Am J Physiol Gastrointest Liver Physiol. 2012;302(10):G1191–G1198. 22403792 10.1152/ajpgi.00252.2011

[108] Abdel-Kawy HS. Effect of carvedilol versus propranolol on acute and chronic liver toxicity in rats. Drug Chem Toxicol. 2021;44(1):101–111. 30810389 10.1080/01480545.2019.1576718

[109] McKee C, Soeda J, Asilmaz E, et al. Propranolol, a β-Adrenoceptor antagonist, worsens liver injury in a model of Non-Alcoholic steatohepatitis. Biochem Biophys Res Commun. 2013;437(4):597–602. 23850676 10.1016/j.bbrc.2013.07.005 PMC5226920

[110] Schepke M, Raab P, Hoppe A, et al. Propranolol stereoisomer plasma concentrations and portal haemodynamic response in patients with liver cirrhosis. Aliment Pharmacol Ther. 1999;13(11):1451–1458. 10571601 10.1046/j.1365-2036.1999.00622.x

[111] Hobolth L, Bendtsen F, Hansen EF, et al. Effects of carvedilol and propranolol on circulatory regulation and oxygenation in cirrhosis: a randomised study. Dig Liver Dis. 2014;46(3):251–256. 24290869 10.1016/j.dld.2013.10.013

[112] Aguilar-Olivos N, Motola-Kuba M, Candia R, et al. Hemodynamic effect of carvedilol vs. Propranolol in cirrhotic patients: systematic review and Meta-Analysis. Ann Hepatol. 2014;13(4):420–428. 24927613

[113] Suna N, Özer Etik D, Öcal S, et al. Effect of propranolol treatment on the incidence of hepatocellular carcinoma in patients waiting for liver transplant with cirrhosis: a retrospective, surveillance study in a tertiary center. Exp Clin Transplant. 2019;17(5):632–637. 31050621 10.6002/ect.2018.0321

[114] Chen Y-C, Li Y-D, Lu C-M, et al. Propranolol use in patients with cirrhosis and refractory ascites: a nationwide study. Saudi J Gastroenterol. 2022;28(2):108–114. 35295067 10.4103/sjg.sjg_586_21 PMC9007076

[115] El-Demerdash E, Abdel-Sattar SA, El-Bakly WM, et al. Antifibrotic effects of carvedilol and impact of liver fibrosis on carvedilol pharmacokinetics in a rat model. Eur J Drug Metab Pharmacokinet. 2017;42(5):767–779. 28012025 10.1007/s13318-016-0391-9

[116] Meng D, Li Z, Wang G, et al. Carvedilol attenuates liver fibrosis by suppressing autophagy and promoting apoptosis in hepatic stellate cells. Biomed Pharmacother. 2018;108:1617–1627. 30372864 10.1016/j.biopha.2018.10.005

[117] Wu Y, Li Z, Xiu AY, et al. Carvedilol attenuates carbon Tetrachloride-Induced liver fibrosis and hepatic sinusoidal capillarization in mice. Drug Des Devel Ther. 2019;13:2667–2676. 10.2147/DDDT.S210797 PMC6681906 31534314

[118] Ling L, Li G, Wang G, et al. Carvedilol improves liver cirrhosis in rats by inhibiting hepatic stellate cell activation, proliferation, invasion and collagen synthesis. Mol Med Rep. 2019;20(2):1605–1612. 31257490 10.3892/mmr.2019.10401 PMC6625452

[119] Bosch J. Carvedilol for portal hypertension in patients with cirrhosis. Hepatology. 2010;51(6):2214–2218. 20513005 10.1002/hep.23689

[120] Mikheeva OM, Drozdov VN, Komisarenko IA. [Pharmacokinetic and pharmacodynamic characteristics of antihypertensive drugs in the treatment of hypertensive patients with chronic diseases of the liver]. Ter Arkh. 2011;83(12):49–55. 22416445

